# The effects of whey protein with or without carbohydrates on resistance training adaptations

**DOI:** 10.1186/s12970-015-0109-4

**Published:** 2015-12-16

**Authors:** Juha J. Hulmi, Mia Laakso, Antti A. Mero, Keijo Häkkinen, Juha P. Ahtiainen, Heikki Peltonen

**Affiliations:** Department of Biology of Physical Activity, Neuromuscular Research Center, University of Jyväskylä, Rautpohjankatu 8, P.O. Box 35, FI-40014 Jyväskylä, Finland

**Keywords:** Hypertrophy, Resistance training, Nutrition, Skeletal muscle, Supplement

## Abstract

**Background:**

Nutrition intake in the context of a resistance training (RT) bout may affect body composition and muscle strength. However, the individual and combined effects of whey protein and carbohydrates on long-term resistance training adaptations are poorly understood.

**Methods:**

A four-week preparatory RT period was conducted in previously untrained males to standardize the training background of the subjects. Thereafter, the subjects were randomized into three groups: 30 g of whey proteins (*n* = 22), isocaloric carbohydrates (maltodextrin, *n* = 21), or protein + carbohydrates (*n* = 25). Within these groups, the subjects were further randomized into two whole-body 12-week RT regimens aiming either for muscle hypertrophy and maximal strength or muscle strength, hypertrophy and power. The post-exercise drink was always ingested immediately after the exercise bout, 2–3 times per week depending on the training period. Body composition (by DXA), quadriceps femoris muscle cross-sectional area (by panoramic ultrasound), maximal strength (by dynamic and isometric leg press) and serum lipids as basic markers of cardiovascular health, were analysed before and after the intervention.

**Results:**

Twelve-week RT led to increased fat-free mass, muscle size and strength independent of post-exercise nutrient intake (*P* < 0.05). However, the whey protein group reduced more total and abdominal area fat when compared to the carbohydrate group independent of the type of RT (*P* < 0.05). Thus, a larger relative increase (per kg bodyweight) in fat-free mass was observed in the protein vs. carbohydrate group (*P* < 0.05) without significant differences to the combined group. No systematic effects of the interventions were found for serum lipids. The RT type did not have an effect on the adaptations in response to different supplementation paradigms.

**Conclusions:**

Post-exercise supplementation with whey proteins when compared to carbohydrates or combination of proteins and carbohydrates did not have a major effect on muscle size or strength when ingested two to three times a week. However, whey proteins may increase abdominal fat loss and relative fat-free mass adaptations in response to resistance training when compared to fast-acting carbohydrates.

## Background

Adequate size and function of skeletal muscle are of paramount importance for health [1–3]. Conversely, excessive fat, especially in the abdominal area, is linked to increased risk of premature death [4] and comorbidities such as negatively altered blood lipid profile [5]. Therefore, it is important to identify lifestyle choices that enhance muscle size and function while concurrently decreasing fat mass, especially in the areas harmful for health.

Resistance training (RT) is the most effective strategy to enhance muscle strength and size, and it may also provide many other health benefits such as enhanced cardiovascular and bone health and functional capacity in daily activities [6, 7]. Of nutritional choices, protein ingestion in the context of a RT bout can enhance skeletal muscle hypertrophy and strength [8, 9]. However, the importance of timing of the protein intake has been questioned lately [10], and possible beneficial effects of post-workout protein nutrition on skeletal muscle has been suggested to be affected by exercise volume, intensity and frequency and the total protein intake of the subjects [9, 11].

Dairy whey proteins seem to promote a reduction of body fat in addition to other potential health benefits [12–14]. In contrast, added sugar, at least in excessive amounts, is linked to increased risk for morbidities and early death [15]. A recent study suggests positive effects of whey proteins on abdominal fat [16], but the effects of whey proteins when compared to carbohydrates in connection with RT are less well known.

Acute protein synthesis and breakdown studies suggest that carbohydrates alone or combination of protein and carbohydrates does not further improve muscle protein balance versus protein alone after single resistance exercise bout when protein alone is sufficient, i.e. at least 20–25 grams [17–19]. However, acute measures after a single exercise bout may not always reflect long-term adaptations to RT [20].Therefore, also long term studies are needed. Bird et al. [21] investigated the effects of added carbohydrates to a small amount of essential amino acid ingestion during resistance exercise bout on RT adaptations. It was found that the combination may be slightly more effective on muscular adaptations than either essential amino acids or carbohydrates alone. This reflects the results of a protein balance study [22] in which added carbohydrates to a small amount of essential amino acids was found to increase protein balance acutely after a resistance exercise bout.

The aim of this randomized, controlled and double-blinded trial was to examine the effects of different post-exercise supplementation regimens on RT adaptation. More specifically, the purpose of this study was to examine the effects of protein and carbohydrate supplementation on body composition and strength as well as blood lipid profile. We hypothesized that proteins alone, along with the combination of proteins and carbohydrates would facilitate a greater increases in muscle size, lean mass and muscle strength with positive effects of whey proteins also on abdominal fat mass and blood lipid profile when compared to isocaloric carbohydrates. The effects of nutritional supplementations were hypothesized to occur independent of the type of RT.

## Methods

### Subjects

A total of 86 healthy, recreationally active men without previous systematic RT background, recruited by newspaper, email list and university web page advertisements, commenced the study. Smokers and those with chronic diseases or prescribed medications, abnormal resting electrocardiography patterns and those training habitually ≥ 2 endurance exercise sessions per week were excluded from the study. The subjects were not allowed to ingest any nutritional supplements during the study other than what were provided, except basic vitamins and minerals.

After comprehensive verbal and written explanations of the study, all subjects gave their written informed consent to participate. The study was conducted according to the Declaration of Helsinki, and ethical approval for the study procedures were granted by the Ethical Committee at the University of Jyväskylä and by the Ethical Committee of the Central Hospital, Jyväskylä.

### Study design

The first phase of the study was a four-week long preparatory RT period, during which subjects were familiarized to RT. This RT period was conducted to standardize training status, to minimize the effects of stressors related to unaccustomed exercise, and to overcome strong neural and learning adaptations known to occur within the first few weeks of RT [23]. In this preparatory RT period, subjects were exercising whole-body workouts two times per week. The subjects used on average nine exercises in one workout, 2–3 sets of every exercise, and 10–15 repetition in every set. Recovery time between the sets lasted two minutes. Training loads were 50–80 % of one repetition maximum (1 RM) increasing throughout the preparatory phase. Bilateral leg press, bilateral knee extension, and bilateral knee flexion exercises were performed during each RT session. The preparatory RT period also included exercises for the other main muscle groups of the body, conducted once a week using machines: chest and shoulders, upper back, trunk extensors and flexors, and upper arms rotated during 2 weekly exercises. Table 1 and 2 lists the main details of the preparatory RT period.

**Table 1:** An overview of the RT program: the first block was a preparatory phase after which supplementations started and within those the subjects were separated into 2 different training regimens. Training bout consisted always of four main exercises trained with the spesific regimen of using either MS, HS or PS as a focus. Five accessory exercises were trained in a HS manner

	Weeks	Training sessions per week	Main aim of training legs BP and LPD		Aim for other accessory exercises	Exercises per session
Prep. period	1–4	2	100 %	ME	ME	9
SHP-group	5–8	2 to 3	75 %	MS	HS	9
			25 %	PS	HS	9
	9–12	2 to 3	25 %	MS	HS	9
			75 %	PS	HS	9
	13–16	2	12.5 %	MS	HS	9
			87.5 %	PS	HS	9
HS-group	5–8	2 to 3	100 %	HS	HS	9
	9–12	2 to 3	75 %	HS	HS	9
	13–16	2	25 %	HS	HS	9
			75 %	MS	HS	9

*ME* muscle endurance, *SHP* Strength-hypertrophy-power training, *HS* Hypertrophy-strength training, *MS* maximal strength, *PS* power & strength, *UFC* until concentric failure, *RM* repetition maximum, *2 to 3* every second week twice per week / thrice per week, *BP* bench press, *LPD* lat pull down

**Table 2:** Typical exercise bout performed 2–3 x week contained exercises for legs, whereas exercises for other muscle groups rotated and thus were trained on average once per week

Exercises in every session:	In HS session:			In MS session:			In PS session:		
	Rest								
Leg press	3–4 × 8–12 or UCF	75–85 % of 1RM	1’	3–5 × 4–6 or UCF	86–95 % of 1RM	3’	3–5 × 3–6	50–80 % of 1RM	3’
Knee flexion	3–4 × 8–12 or UCF	75–85 % of 1RM	1’	3–5 × 4–6 or UCF	86–90 % of 1RM	3’	3–5 × 3–6	50–80 % of 1RM	3’
Knee extension	2–3 × 10–15 or UCF	75–85 % of 1RM	1’	3–5 × 4–6 or UCF	86–90 % of 1RM	3’	3–5 × 3–6	50–80 % of 1RM	3’
Accessory exercises rotated between session I and II:									
Bench press / LPD	3–4 × 10–15 or UCF	70–85 % of 1RM	1’	3–5 × 4–6 or UCF	86–95 % of 1RM	3’	3–5 × 3–8	50–80 % of 1RM	3’
Other exercises	2–4 × 8–15 or UCF	70–85 % of 1RM	1’	2–4 × 8–15	70–85 % of 1RM	2’	2–4 × 8–15	70–85 % of 1RM	2’
Exercises in every 2nd session									
Session I: main exercise: bench press. Other exercises: shoulder press, elbow extensors, upper-back/rear deltoideus, hip abductors and adductors.									
Session II: main exercise: lat pulldown. Other exercises horizontal row, elbow flexors, torso rotators, abdominals, back extensions.									

*ME* muscle endurance, *SHP* Strength-hypertrophy-power training, *HS* Hypertrophy-strength training, *MS* maximal strength, *PS* power & strength, *UCF* until concentric failure, *RM* repetition maximum, *2 to 3* every second week twice per week / thrice per week, *LPD* lat pull down

Before randomization further into different intervention groups, eight subjects declined to continue with the study during the preparatory RT period. This resulted in 78 subjects (age 34.4 ± 1.3 years, height 1.80 ± 0.08 m, weight 83.6 ± 1.4 kg) who started the actual RT program with different supplementary nutrition. These subjects were randomized into three groups: whey protein (*n* = 25), carbohydrates (CHO, *n* = 25) or whey protein + carbohydrates (*n* = 28). The variation in the responses to body composition and strength was hypothesized to be larger in the combination group than in the protein or carbohydrate groups, so the n size was slightly larger in that group at the start. Within these groups, the subjects were further divided into two different RT regimens: 1) training aiming especially for muscle hypertrophy and strength (HS) and 2) training aiming especially for muscle strength, hypertrophy and power (SHP) for 12 weeks. Subjects were advised to continue their normal recreational physical activities such as low-intensity walking, skiing, cycling and swimming during the study.

### Resistance training protocols

Whole-body RT that started after the preparatory RT period was undertaken 2–3 times per week, depending on the phase of the training program, for a total of 28 training sessions. Table 1 and 2 lists the main details of the RT period. The training techniques were carefully supervised and the training was controlled throughout the whole RT period. The individual loads were determined by the strength tests (repetitions to failure: 2–6RM) for all main exercise during the first week of each 4-week training block using the Brzycki formula [24]. The loads were then adjusted throughout the training in each training block. The sets were conducted to a last possible repetition that could be performed with good technique or until concentric failure. The exception to this were the power-strength (PS) sets that were conducted with maximal concentric speed and, thus, not close to concentric failure. The sets, repetitions and loads fluctuated throughout each training block in a modern manner using aspects from block and non-linear periodization [25, 26]. This is important as training variety is crucial for stimulating further development in muscle strength after the first few weeks of training [26]. However, a general long-term plan was to increase absolute and relative (%-1RM) loads in a progressive manner with a short peaking period at the end of each training block before the outcome measurements.

The following exercises were used in each training session: bilateral leg press, knee extension, and knee flexion. The training program also included exercises for the other main muscle groups of the body: chest and shoulders, upper back, trunk extensors and flexors, and upper arms conducted every second training session. Hypertrophy-focused strength (HS) training contained mainly sets of 8–12 repetitions with 75–85 % loads of 1 RM. Maximal strength (MS) training in both RT regimens consisted of neural enhancing RT with lower repetitions per set (typically 4–6) and higher intensity (86–95 % 1 RM), but also more traditional hypertrophy sets to increase muscle size. PS training consisted of sets with lower loads of 1 RM (50–80 % 1 RM) performed with maximal concentric speed.

To shortly describe the RT program, the 12-week periodized RT was divided further into three different blocks. Every block consisted of four weeks of RT. In the first block, SHP group had 25 % power-strength (PS) and 75 % maximal-strength (MS) training sessions, in the second 75 % PS and 25 % MS training sessions and in the last 87.5 % PS and 12.5 % MS training sessions.

By contrast, in HS training groups, the first block consisted of 100 % HS sessions, in the second block 75 % HS and 25 % MS training sessions and in the last block 25 % HS and 75 % MS of the total training sessions per block. This type of RT program has been used in previous studies in our lab [27], and it is in line with the American College of Sports Medicine (ACSM) position stand [28] recommendations of progression models in RT.

Thus, in short, the main difference between these two training regimens (SHP vs. HS) was that in SHP power-strength sets replaced part of the hypertrophy-focused sets, especially at the end of the training program and therefore the volume of sets aiming for maximal hypertrophy was higher in HS than in SHP.

### Nutritional supplementation during resistance training

During the 12-week RT intervention, pre-sweetened post-workout supplements were mixed in 0.5 L water and consumed immediately following every training bout in a double blind fashion. One group received protein, one group carbohydrate, and one group protein plus carbohydrate. Protein and carbohydrates were provided by Northforce (Kuusamon Juusto Oy, Kuusamo, Finland). Protein group received 37.5 grams of whey concentrate (30 g of whey proteins, 5 g of lactose < 1 g of fat) and carbohydrate group received 34.5 grams of maltodextrin being thus isocaloric to whey protein. In contrast, protein plus carbohydrate group received 37.5 grams of whey concentrate (30 g of whey proteins) and 34.5 grams of maltodextrin. The supplements were mixed with non-caloric sugar-free drinks (FUN Light provided by Orkla Foods Finland, Turku, Finland) depending on the week and subject’s preference (either strawberry, forest fruit, pomegranate-strawberry, apple-pear or raspberry-lemon). The subjects were advised to eat normal recommended mixed meal based on the Finnish Nutrition Recommendations 2014 (see below) 1–2 hours after the exercise bout.

### Daily nutrient intake

Subjects kept 4-day food diaries during the second block of the 12-week RT period. Dietary intake was recorded over three weekdays and one weekend day. The researchers gave subjects both verbal and written nutritional recommendations based on the Finnish Nutrition Recommendations 2014. As a rule, these follow the recommendations for the Nordic countries in Europe published in Autumn 2013 (NNR2012) and are very close to USDA and HHS dietary guidelines (2010) for normal healthy adults. The subjects were instructed on how to report nutritional intake in the diaries. Nutrients provided by the supplements were included in the analysis. The food diaries were analyzed by nutrient analysis software (Nutri-Flow; Flow-team Oy, Oulu, Finland).

### Body composition

Body composition was estimated by Dual-energy X-ray absorptiometry (DXA, Lunar Prodigy Advance, GE Medical Systems – Lunar, Madison WI USA) before the preparatory RT period, before the supplementations started and after the experimental RT. DXA measurements were conducted following a 12-hour overnight fast and 24-h absence of alcohol and strenuous exercise. Subjects were tested on their back in a supine position on the DXA table with their arms at their sides and feet together with minimal clothing (i.e., a pair of shorts). Legs were secured by non-elastic straps at the knee and ankles, and the arms were aligned along the trunk with the palms facing the thighs. All metal objects were removed from the subject before the scan. Analyses (using enCORE 2005, version 9.30 and Advance 12.30) provided total, lean (including muscle) and fat masses. The same investigator conducted all the analyses. Automatically generated regions of the legs were manually adjusted by the same investigator to include the hamstrings and gluteal muscles. Thus, legs were separated from the trunk by a horizontal line right above the iliac crest providing lean and fat mass for legs and upper body separately. In fat-free mass (FFM) excluding bones, the present study focuses on total and leg mass as also the other measurements (muscle CSA and muscle strengths) in the current study are from the legs. The results are presented as absolute measures and as normalized to total body mass. The trunk region includes the neck, chest, abdominal and pelvic areas except the gluteal area that was included into legs. The android region is the area between the ribs and the pelvis within the trunk region (the upper part of the trunk). This area correlates with visceral fat measures [29] and is highly associated with metabolic abnormalities [30] and, thus, was selected for the present investigation. These customized range of interests were then copied to the DXA scans obtained at weeks 0 and 12 to assure that analyses were conducted from the same areas at all measurement times. In a previous study in our laboratory an intraclass correlation coefficient (ICC) for the body composition measures were 0.786–0.975 [31].

### Muscle cross-sectional area

Cross-sectional area (CSA) of the knee extensor muscles at the mid-thigh (vastus lateralis, rectus femoris, and vastus intermedius) were measured by the extended field of view mode using a B-mode axial plane ultrasound (model SSD-2000, Aloka, Tokyo, Japan) with a 10-MHz linear-array probe. A customized convex-shaped probe support coated with water-soluble transmission gel was used to assure a perpendicular measurement and to constantly distribute pressure on the tissue. The measurements were conducted twice: before the supplementations started and after the experimental RT. The transducer was moved manually from lateral to medial along a marked line on the skin. Panoramic cross sectional images were conducted at 50 % of the femur length (lateral aspect of the distal diaphysis to the greater trochanter), and CSA was analysed manually using ImageJ software (version 1.44p; National Institutes of Health, Bethesda, MD). Each leg extensor muscle CSA was analysed three times. The two closest values for each muscle were averaged, summed for total knee extensor CSA, and this value was used for statistical analyses. The method has been shown to be very reliable and valid against magnetic resonance imaging (MRI) to detect RT-induced change in muscle size in our laboratory, e.g. ICC > 0.9 and high limits of agreement by Bland Altman method [32].

### Maximal strength testing

Maximal strength was measured before the 4-week preparatory RT period, after the preparatory RT period and thus before the supplementation started, and after the 12-week experimental RT period. In addition, the subjects came to the laboratory once before the study began to learn the techniques in the strength test devices. Isometric strength was already then performed maximally to investigate the reliability of the testing between this preliminary session and the actual pre-test session in these subjects. The analysis of reliability revealed an ICC of 0.945 for isometric strength measurement.

In the actual measurements, the subjects were carefully familiarized with the test procedures and had several warm-up contractions on all devices. A David 210 horizontal leg press device (David Health Solutions Ltd, Finland) was used to measure maximal bilateral dynamic concentric strength of the leg extensors (hip and knee extensors). In the actual test, the subjects had as many trials as required to determine 1 RM. Between the trials, subjects were allowed to rest for one minute in the first light weights and thereafter two minutes when the maximal weights were approached. The device was set up so that the knee angle in the initial flexed position was on average 60° and a successful trial was accepted when the knees were fully extended (approximately 180°). The greatest load that the subject could lift to full knee extension was accepted as 1RM.

In addition, a horizontal leg press extension dynamometer (Department of Biology of Physical Activity, University of Jyväskylä, Jyväskylä, Finland) was used to determine maximal isometric bilateral leg press force (maximal voluntary contraction, MVC). Subjects were seated with a hip and knee angle of 110° and 107°, respectively, and were instructed to produce maximal force on verbal command and to maintain the force plateaued for 3–4 s. In total, 3 maximal trials with one minute rest were performed. At least three trials separated by a rest period of 1 minute or more when needed were conducted, and up to two additional trials were performed if the maximum force during the last trial was greater by 5 % compared with that during the previous attempt. The trial with the highest maximal force measured was used for statistical analysis.

### Venous blood sampling and analysis

Venous blood samples were collected before the preparatory RT period and every four weeks thereafter. Venous blood samples were drawn after 12 h of fasting to obtain concentrations of total cholesterol, LDL, HDL and triglycerides. Subjects were asked to rest for at least 8 h during the preceding night and were required to refrain from strenuous physical activity for at least 48 h. Blood samples were taken from the antecubital vein into serum tubes (Venosafe; Terumo Medical Co., Leuven, Hanau, Belgium) using standard laboratory procedures. Blood samples were stored in room temperature for 10 min, after which they were centrifuged at 3500 rpm for 10 minutes (Megafure 1.0 R Heraeus; DJB Lab Care, Germany) and the serum obtained was immediately analyzed by spectrophotometry (Konelab 20XTi; Thermo Fisher Scientific, Vantaa, Finland). LDL concentration was estimated using the Friedewald [33] equation: LDL = total cholesterol - HDL - (triglycerides/2.2).

### Statistical analysis

All data are expressed as means ± SE, except where designated. The data were analysed by a repeated measures General Linear Model ANOVA and using time and nutrition as factors with training type as a covariate when appropriate. Possible training-type x nutrition x time interactions were analysed using a 3-factor repeated measures General Linear Model ANOVA. Any violations of the assumptions of sphericity were explored and, if needed, corrected with a Greenhouse-Geisser (if estimated epsilon (ε) is < 0.75) or Huynh-Feldt estimator (if estimated epsilon (ε) is ≥0.75). The differences in the changes from pre to post measurements between different supplement groups were analysed using univariate ANOVA and training type (HS or SP) as a covariate. Bonferroni post hoc tests were performed to localize differences between and within the treatments and/or time-points. For the data that was not normally distributed, a non-parametric Wilcoxon signed rank test was used. SPSS version 13.0 for Windows was used for statistical analyses (SPSS, Inc., Chicago, IL). The level of significance was set at *P* < 0.05.

## Results

There were no differences among the groups in the rate of noncompliance or drop-outs (carbohydrates, *n* = 4, protein, *n* = 3, protein + carbohydrates, *n* = 3). Baseline physical characteristics of the subjects (*n* = 68) who completed the different supplemental and training programs are presented in Table 3.

**Table 3:** Characteristics of subjects after the habituation before the actual 12 -week RT interventions started and average daily dietary intakes from four-day diary during the second four-week training block

	CHO (*n* = 21)	Protein (*n* = 22)	Protein + CHO (*n* = 25)	All (*n* = 68)
Age (y)	36.4 ± 4.2	31.4 ± 1.4	36.2 ± 1.2	34.7 ± 1.4
Height (m)	1.79 ± 0.02	1.81 ± 0.02	1.80 ± 0.02	1.80 ± 0.01
Weight (kg)	81.4 ± 2.5	83.8 ± 2.4	85.1 ± 2.3	83.6 ± 1.4
Energy (kJ/kg/day)	146.5 ± 8.4	124.0 ± 10.3	122.5 ± 8.9	129.4 ± 5.5
Protein (g/kg)	1.7 ± 0.1	1.5 ± 0.1	1.4 ± 0.1	1.5 ± 0.1
Protein (%)	20.0 ± 0.6	21.2 ± 1.1	20.2 ± 1.1	20.5 ± 0.6
Fat (g/kg)	1.4 ± 0.1	1.1 ± 0.1	1.1 ± 0.1	1.2 ± 0.1
CHO (g/kg)	3.5 ± 0.3	3.0 ± 0.3	3.0 ± 0.3	3.2 ± 0.2
HS (n)	10	13	14	37
SP (n)	11	9	11	31

Data are means ± SE. There were no significant differences between the groups. FFM = fat-free mass, CHO = carbohydrates. HS = hypertrophic-strength training and SP = strength-and power training. The nutrition results also include the supplement that was ingested for 1 or 2 days during the four day diary recording.

### Preparatory RT period

**Fig. 1:**
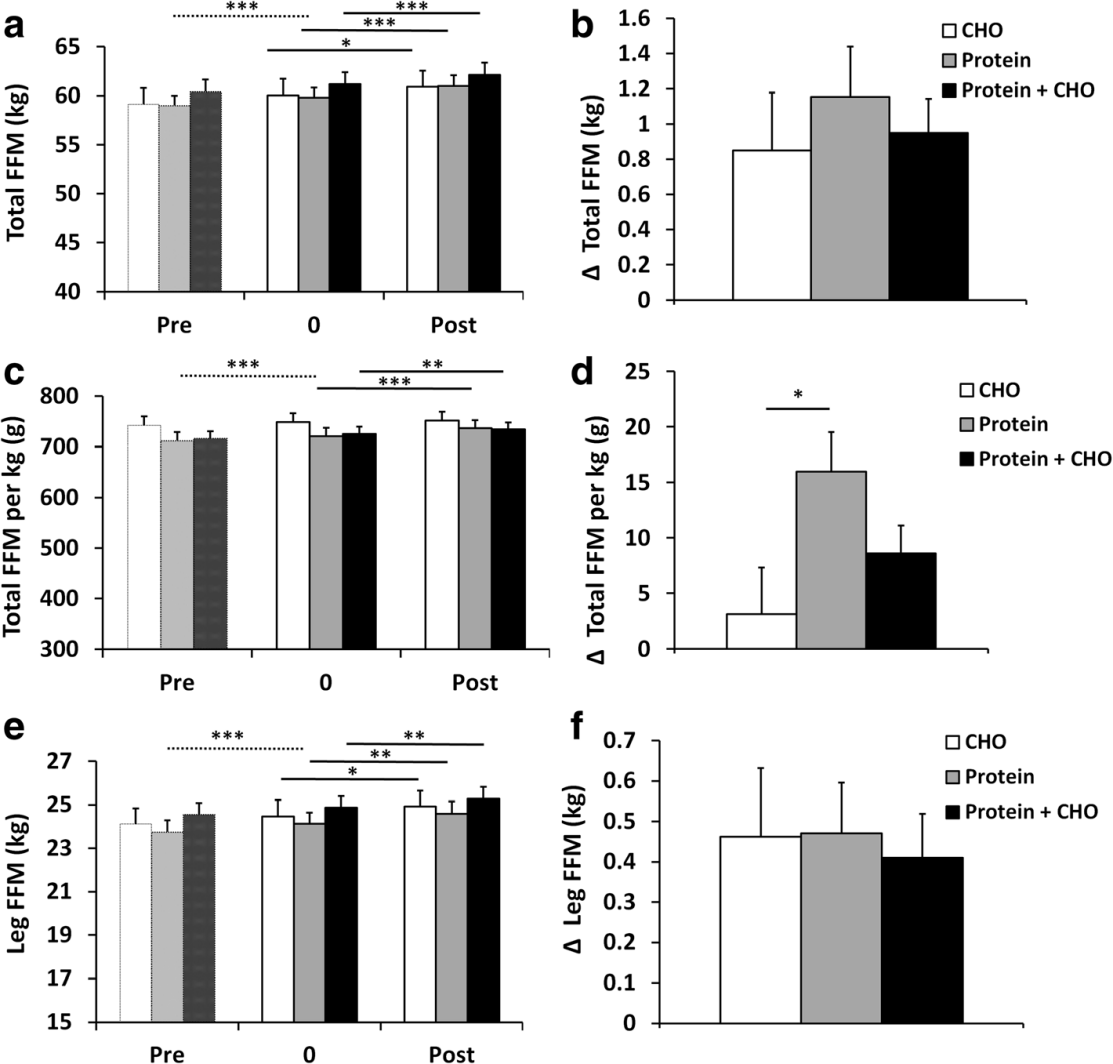
**a** Total fat-free mass (FFM), (**b**) total FFM changes, (**c**) relative FFM (total FFM divided by the body weight), (**d)** relative FFM changes, (**e**) leg FFM, and (**f**) leg FFM changes. The changes are from the beginning of supplementation (week 0) to the end of the training period (week 12) in carbohydrate (CHO), protein, and protein and carbohydrate groups. * *p* < 0.05, ** *p* < 0.01, *** *p* < 0.001 depict significant differences. During the preparatory RT period the difference to the week 0 is analyzed as one group and depicted using dashed line as no supplementation was provided before the week 0

**Fig. 2:**
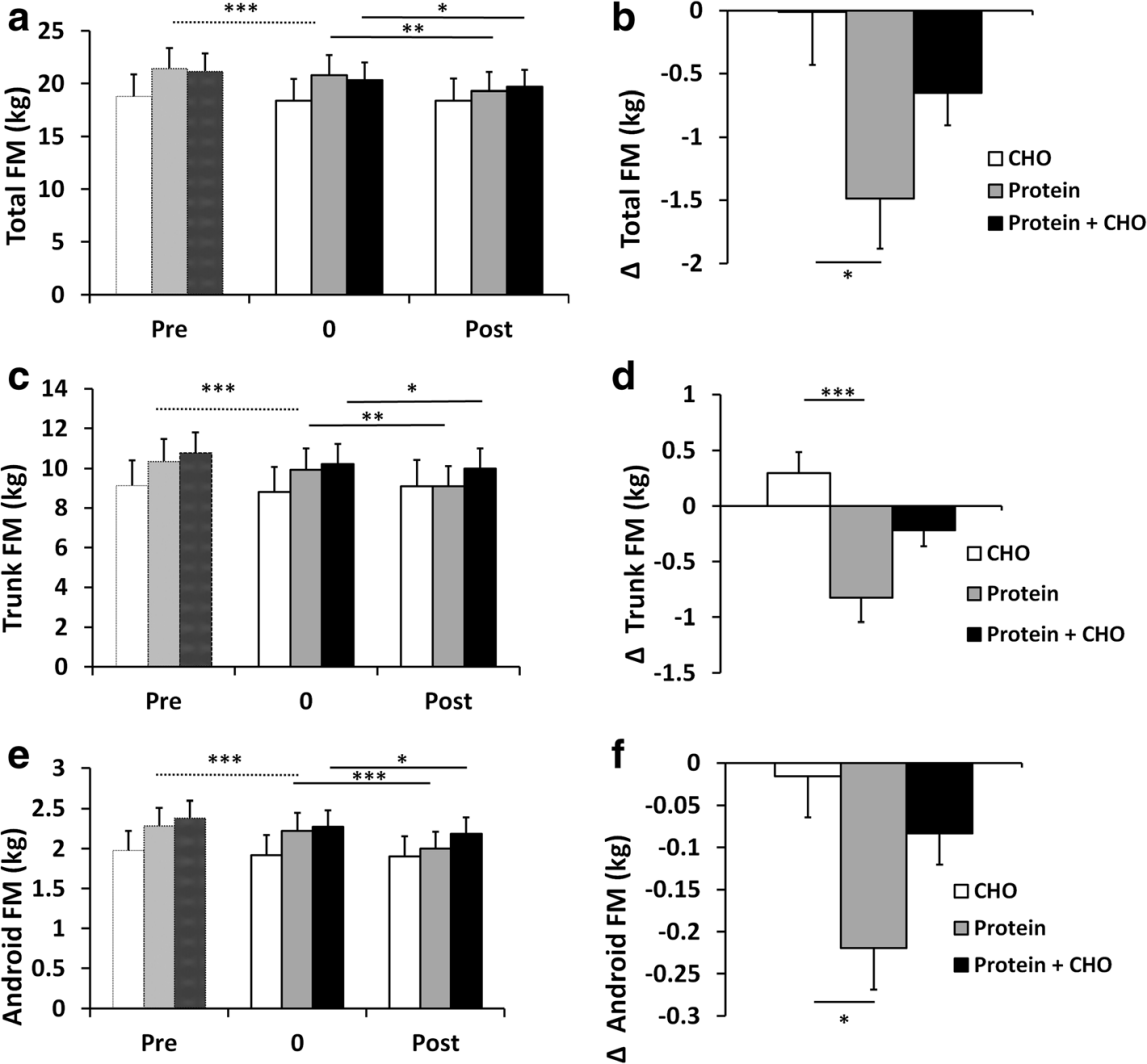
Total fat mass (**a**), total fat mass changes (**b**), trunk fat mass (**c**), trunk fat mass changes (**d**), android fat mass (**e**), android fat mass changes (**f**) in carbohydrate (CHO), protein, and protein and carbohydrate groups. * (*p* < 0.05), ** (*p* < 0.01), *** (*p* < 0.001) depict significant differences within each treatment (**a**, **c**, **e**) or between the treatments (**b**, **d**, **f**).

**Fig. 3:**
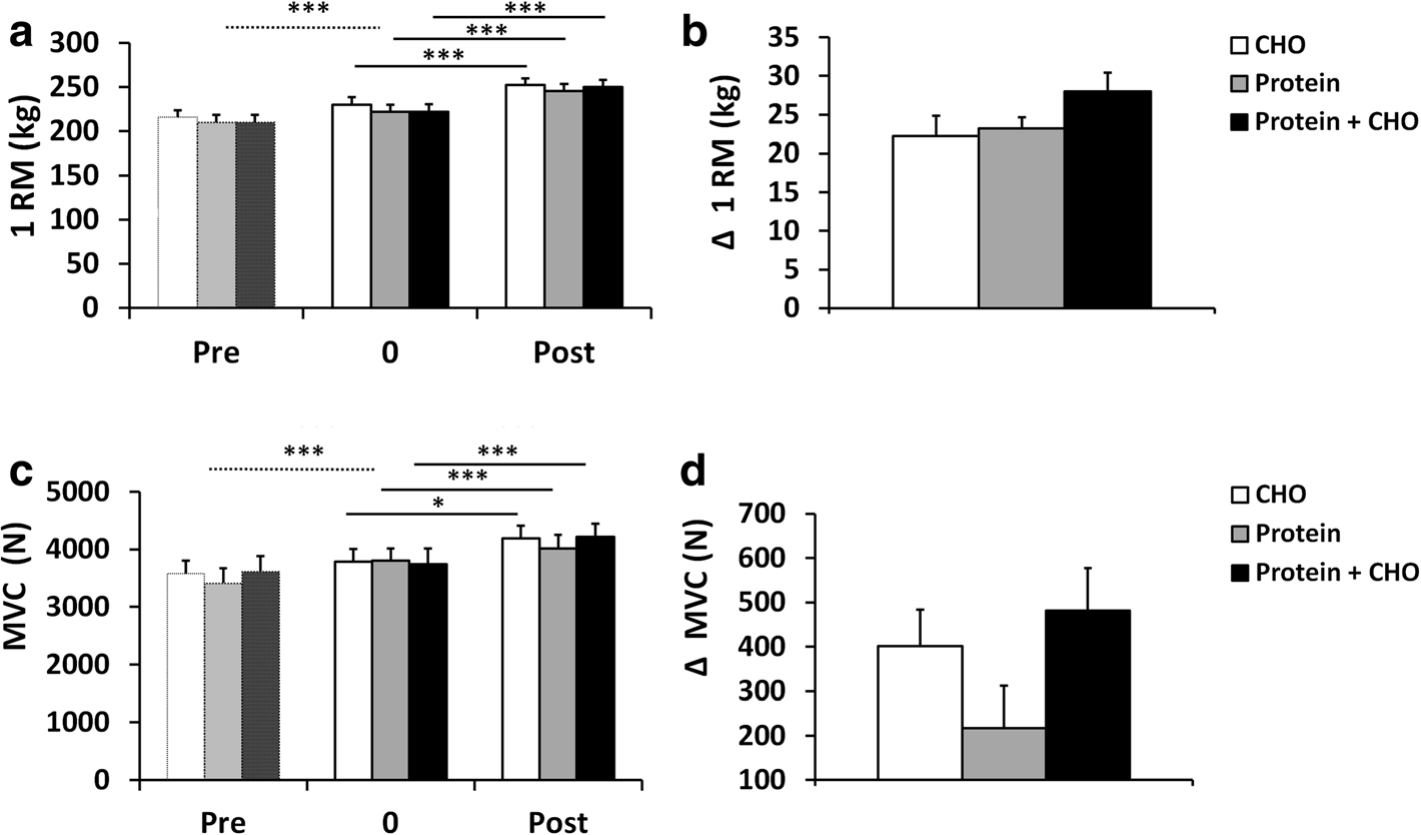
Maximal dynamic strength 1RM (**a**), changes in 1RM (**b**), isometric strength (MVC) (**c**) and changes in isometric strength (MVC) (**d**) in carbohydrate (CHO), protein, and protein and carbohydrate groups. * *p* < 0.05, *** (*p* < 0.001) depict significant differences within each treatment (**a**, **c**, **e**) or between the treatments (**b**, **d**, **f**).

The 4-week preparatory RT period was used to standardize the training background of the subjects. This short RT period increased FFM (total and in legs) (*P* < 0.001) (Fig. 1). Total body, trunk, android (*P* < 0.001, Fig. 2) and leg fat masses (not shown, *P* < 0.05), all decreased. Muscle strength (1 RM and MVC) increased (*P* < 0.001) (Fig. 3). Of serum lipids, total cholesterol decreased after the preparatory RT period (*P* = 0.001) (Table 4). There were no differences between the groups later randomized into different supplement groups.

**Table 4:** Blood lipids

	CHO (*n* = 21)	Protein (*n* = 22)	Protein + CHO (*n* = 25)	All (*n* = 68)
S-Chol Pre				5.00 ± 0.12**
S-Chol 0	4.70 ± 0.21	4.61 ± 0.19	4.98 ± 0.20	4.77 ± 0.18
S-Chol 12	5.02 ± 0.22	4.52 ± 0.17	5.07 ± 0.24	4.87 ± 0.13
S-HDL Pre				1.45 ± 0.05
S-HDL 0	1.46 ± 0.08	1.46 ± 0.08	1.48 ± 0.07	1.47 ± 0.04
S-HDL 12	1.39 ± 0.10	1.33 ± 0.07	1.37 ± 0.07	1.36 ± 0.05
S-LDL Pre				3.12 ± 0.10
S-LDL 0	2.98 ± 0.18	2.87 ± 0.19	3.24 ± 0.18	3.04 ± 0.11
S-LDL 12	2.89 ± 0.21	2.65 ± 0.14	3.09 ± 0.20	2.88 ± 0.11
S-Trig. Pre				1.20 ± 0.09
S-Trig. 0	1.08 ± 0.16	1.05 ± 0.08	1.12 ± 0.11	1.09 ± 0.07
S-Trig. 12	1.40 ± 0.21**	1.20 ± 0.11*	1.30 ± 0.15	1.30 ± 0.09**

Data are mean ± SE (mmol/L). *Trig* triglycerides / triacylglyrerols. * (*p* < 0.05), ** (*p* < 0.01), *** (*p* < 0.001) depict significant differences from the representative 0-time-point. Note that even though from weeks 0 to 12 there was an increasing trend in all the groups, resistance training from Pre to week 12 (16 weeks in total) did not have significant effect on blood triglycerides

### Training type

After the preparatory RT period, the subjects within all three supplementation groups trained with either the hypertrophic-strength (HS) or strength-hypertrophy-power (SHP) focused program for 12 weeks. Muscle strength and size increased and fat mass decreased in both training groups (*P* < 0.05). The comparison between the training types *per se* is not the focus of the present study concentrating on the three groups of supplemental nutrition. There were no nutrition x training-type x time interaction effects on any variables investigated (*P* > 0.05). This means that the type of RT did not have an effect on the nutrition responses. Therefore in the following figures and results, the two different training types are shown as pooled. However, to minimize even the small possible effects of the training type, the statistics were always conducted with the training type (HS or SHP) as a covariate.

### Daily nutrient intake

All three groups reported to consume approximately 20 E% proteins and 40 E% carbohydrates, which was slightly high for protein and low for carbohydrates (10–20 % of proteins and 45–60 % of carbohydrates). Although the protein group tended to have lower energy intake (*P* = 0.1), the dietary intake did not differ significantly between the groups when expressed relative to body weight (Table 3).

### Body composition

#### Fat-free mass

Significant increases following RT for all three supplemental groups were seen for total FFM (*P* < 0.001) and leg FFM (*P* = 0.001) (Fig. 1). There were no differences in the changes between the different supplemental groups for the absolute FFM changes. However, the protein group increased relative FFM (per kg bodyweight) more than the carbohydrate group (*P* < 0.05) (Fig. 1d).

#### Fat mass

Total fat mass (FM) (*P* = 0.001) (Fig. 2) and leg FM (*P* = 0.002) (not shown) decreased following RT. Leg FM decreased similarly in all nutrition groups (no nutrition x time interaction: *P* = 0.302). However, total FM showed a nutrition x time interaction effect (*P* = 0.032). This was seen as a decrease following RT in the protein (*P* = 0.001) and protein + carbohydrate (*P* = 0.02) groups, but not in the carbohydrate alone group (*P* = 0.98) (Fig. 2). This change in total FM (*P* = 0.03) was also larger in the protein group compared with the carbohydrate group without differences in the leg FM (*P* = 0.427).

Trunk FM was unchanged following RT (*P* = 0.07) whereas android FM decreased due to RT (*P* < 0.001) (Fig. 2). A nutrition x time interaction was detected for trunk FM (*P* = 0.001) and for android FM (*P* = 0.011). Both trunk (*P* = 0.001 and *P* = 0.001) and android (*P* < 0.001 and *P* = 0.02) FM decreased following RT in the protein and protein + carbohydrate groups, respectively (Fig. 2). A post hoc test showed that these changes in trunk and android FM were larger in the protein group compared to the carbohydrate group (*P* < 0.001 and *P* = 0.01), respectively (Fig. 2).

### Muscle size

**Fig. 4:**
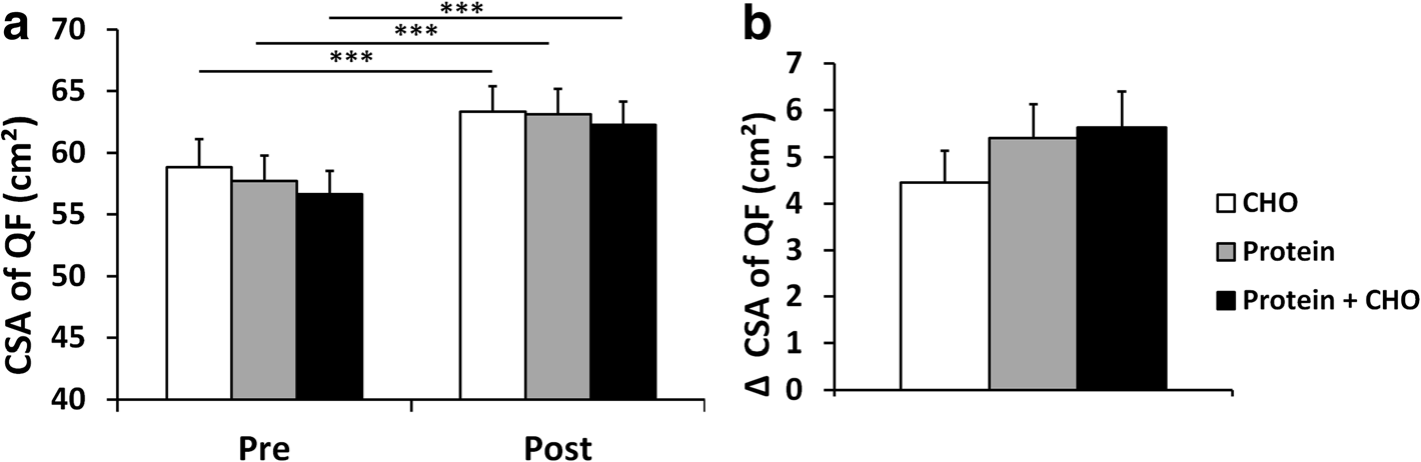
Cross sectional area (CSA) of leg extensor muscles (quadriceps femoris, QF) excluding (vastus medialis muscle) (**a**) and absolute changes in CSA (**b**) in carbohydrate (CHO), protein, and protein and carbohydrate groups. *** (*p* < 0.001) depict significant differences within each treatment (**a**, **c**, **e**).

The CSA of leg extensor muscles increased following RT (P < 0.001) without nutrition x time effects (*P* = 0.715) (Fig. 4). Thus, CSA increased in all supplemental groups (*P* < 0.001).

### Maximal strength

Significant increases following RT were seen for 1 RM (*P* < 0.001) and for isometric strength (*P* < 0.001) of leg and hip extensor muscles (Fig. 3). No nutrition x time interaction effects were observed for 1 RM strength (*P* = 0.360) and for isometric strength (*P* = 0.129).

### Blood lipid profile

Serum lipids were measured every 4 weeks. Total cholesterol (*P* = 0.753), HDL (*P* = 0.162), LDL (*P* = 0.110) or triglycerides (*P* = 0.433) did not show significant overall RT effect from the beginning of the training, i.e. 16 weeks of training (Table 4). No nutrition x time interaction effects were observed for total cholesterol (*P* = 0.126), HDL (*P* = 0.953), LDL (*P* = 0.476) and for triglycerides (*P* = 0.752).

## Discussion

The purpose of this study was to investigate the effects of postexercise protein and carbohydrate supplementation alone or in combination on RT adaptations. Significant increases following RT were observed for quadriceps muscle cross-sectional area (~9 %), total body FFM (~2 %) and muscle strength (~10-11 %) with only marginal effects of the supplemental nutrition. However, postexercise whey protein intake reduced total (~6 % vs. 0 %) and abdominal (~8 % vs. 0 %) fat when compared to carbohydrates (fast acting glucose polymers) supplementation, respectively. This led to an increased relative FFM change in the protein (~2.5 %) when compared to the carbohydrate group (~0.5 %), independent of the type of RT. These results were not accompanied by changes in serum lipid profile.

Adequate size and function of skeletal muscle [1–3] and rather low fat mass in the abdominal areas are of paramount importance for health [4]. The only significant effect of the supplements observed in the present study on lean or muscular tissue was the larger relative gains of FFM in the protein group when compared to the carbohydrate group. This was driven by the significantly larger decrease in fat mass and non-significantly higher increase in FFM by the protein group. Therefore, more positive body composition changes may be achieved with post-exercise ingestion of whey proteins when compared to isocaloric carbohydrates. Previous studies have shown that protein ingestion can enhance skeletal muscle hypertrophy and strength in response to chronic RT [8, 9]. Whey contains high quality proteins [34] which have increased muscle CSA adaptation to RT even in subjects ingesting 1.4–1.5 g/kg body weight of protein in their daily nutrition [27]. However, not all studies have found positive effects of protein ingestion and the importance of timing of the protein intake and the post-exercise intake of protein *per se* has been questioned lately [10]. Indeed, the possible beneficial effects of the post-workout protein nutrition may be affected by at least the volume, intensity and frequency of training and of the nutritional state of the subjects [9, 11]. In the present study, however, the type of training did not have major influence on the effects of the supplements. Previously, Farup et al. [35] observed improved muscle size gains by whey protein when compared to carbohydrates independent of training type (eccentric vs. concentric RT). More studies are needed to investigate the effects of nutrition in different types of resistance training modalities in the future studies.

No effects of supplementation were observed on muscle strength. The reason for a lack of change may be due to small differences in muscle size and also the fact that increased muscle strength during the first months of RT is achieved through not just increased muscle size, but especially through neural adaptations [23] that may be less responsive to nutrition. This may have occurred, even though we had a 4-week preparatory RT period to accommodate the influence of neural adaptations on muscle strength as has been suggested [9, 36]. Thus, neural and possibly other confounding factors and high individual variation on muscle strength adaptation [37] may be the reason why the effects of postexercise nutrient supplementation have been less consistent for muscle strength adaptation than for muscle hypertrophy [9].

In addition to protein vs. carbohydrate comparison, an important aim of the study was to investigate the effects of adding carbohydrates to the postexercise drink. Acute protein synthesis and breakdown studies suggest that the addition of carbohydrates does not further improve muscle protein balance versus sufficient ingestion of protein alone acutely after a single resistance exercise bout [17–19]. The present study also supports this evidence showing that adding carbohydrates to a protein drink did not enhance muscular adaptation to RT. Previously, Bird et al. [21] investigated the effects of added carbohydrates to a small amount of essential amino acid (total 6 g) ingestion divided into small doses ingested between each set of resistance exercise bout. They reported that the combination may be slightly more effective on muscular adaptations than the choices alone. This supports a protein balance study also using small amount (~6 g) of essential amino acids [22]. Clearly, more long-term training studies are needed to investigate this phenomenon.

Long-term RT can provide benefits to body composition such as improved muscle mass and decreased fat mass [6], which were also observed in the present study. In addition to RT, many studies support replacing dietary carbohydrates or fats with dietary protein for favorable changes on decreasing fat mass [38, 39]. However, the effect of whey protein supplementation during RT on fat mass are conflicting [40]. Volek et al. [41] demonstrated that whey protein supplementation did not promote fat loss more than carbohydrate supplementation during RT. However, Cribb et al. [42] reported that fat mass decreased in a group consuming whey proteins during 10 weeks of RT. Moreover, a study by Arciero et al. [16], although lacking a placebo group, suggests that whey alone and whey protein combined with RT reduces total fat, abdominal fat and visceral fat mass. This is consistent with the results of the present study, where total, trunk and android fat of the whey protein group reduced when compared to the carbohydrate group. Interestingly, unlike the total and trunk area fat, however, the leg fat mass that decreased after RT, was not affected by the supplemental nutrition. Recently, Antonio et al. [43] investigated the effects of a very high protein ingestion (on average 3.4 g/kg per day) during 8 weeks of RT. Whey or beef protein powder was provided for the subjects to supplement their normal meals so that they achieve this high level of protein ingestion. The adaptations where compared to a group with rather high protein ingestion (2.3 g/kg per day). The result was that the very high protein group lost an average of 1.6 kg of fat mass when compared to only 0.3 kg in the high protein group. These studies combined suggest that supplementary whey protein ingestion can decrease fat mass during RT when ingested in the context of a resistance exercise workout or throughout the day.

The present study did not have a RT only group so we can only speculate whether the supplementary carbohydrate ingestion blocked the effects of RT on fat mass loss or whether whey proteins potentiated or maintained the fat mass loss of RT in the present study. Nevertheless, whey protein may have either decreased energy intake and/or increased energy expenditure when compared to the carbohydrate group in the subjects with a written and verbal recommendations to follow the Nordic recommendations published in Autumn 2013 (NNR2012). Indeed, although not significant, total energy intake tended to be lower in the whey protein group compared to the carbohydrate group (*P* = 0.1). The known effect of dairy proteins on satiety and decreased energy intake [13, 44] may, in part, explain why the whey protein group showed decreased fat mass when compared to carbohydrates. In addition to a rather short 12-week length of the study, this slightly lower macronutrient intake and not higher total protein intake may also explain why in the absolute terms the whey group did not increase muscle size and strength more that carbohydrates alone, only relative FFM [11]. Another potential reason that there was no observed increase in FFM or muscle CSA compared to carbohydrates alone was that the subjects only took supplements after workouts, i.e. 2–3 times per week. In addition to energy intake, whey proteins have been reported to increase postexercise resting energy expenditure (REE) when compared to carbohydrates [45] or non-energy placebo [46], up to 24 hours [45]. Whey proteins have been also shown to increase fat oxidation [47] and lipolysis [48] when compared to carbohydrates and also markers of lipolysis directly in visceral fat pad at least in rodents [49]. Therefore, it is speculated that both energy intake and expenditure were affected in the whey protein group contributing to the ~1 kg larger decrease in total and 0.2 kg of abdominal / android fat mass when compared to the carbohydrate group. The beneficial effects of whey proteins on abdominal fat were not, however, associated with altered blood lipid profile. Previously, dairy whey proteins have been shown to have various health benefits [12–14] in contrast to excessive amounts of added sugar [15]. Future studies should investigate in humans whether a form of whey proteins (e.g. intact vs. hydrolyzed) also may have an effect on body fat and muscle masses and their regulation as may be suggested based on recent rodent studies [49, 50].

Interestingly, the replacement of carbohydrates by whey protein did offer benefits to fat mass decrease, but when whey was added to carbohydrates, the result was in between the carbohydrate and whey group. Indeed, a meta-analysis [40] suggests that whey when consumed as a replacement, not as a supplement, may decrease fat mass. It is possible that in a study with *ad libitum* diet, such as the present one, whey protein ingestion alone may offer benefits for the athlete if he/she wants to decrease fat mass. However, it can be speculated that if the main goal is to increase muscle and body mass, one has to be careful to potentially eat more when ingesting these high satiating, REE-inducing proteins, otherwise the energy intake may be too low for optimal adaptations and recovery, at least in some individuals.

The major strengths of the present study were the relatively large number of subjects, two different types of RT and perhaps especially, a preparatory RT period at the start. Most of the training and nutrition studies are conducted in previously untrained subjects, which is problematic as the stressors related to unaccustomed exercise may potentially confound interpretation of the true effects of different types of training or even nutrition and the neural effects can be overriding the effects of muscle mass [23]. We believe that this strategy should be used more in the future studies as well.

A limitation of our study is that we only had one time point for the dietary diaries. By having a dietary diary also before the study period we could have directly assessed the effects of different supplemental groups on changes in daily dietary intake. We were also not able to get diaries from the last weeks of the study due to the already very demanding study for the subjects. Due to a careful randomization and such a large n-size, we find, however, it very improbable that there would have been consistent differences between the groups by a chance alone. Furthermore, DXA measures the total fat of the entire region of interest and thus both visceral and subcutaneous. However, the upper abdominal android region measure of DXA well correlates between visceral fat measured by computed tomography (CT) scan (R = 0.78) [29] and the response of trunk/abdominal and visceral fat masses to RT and protein nutrition have been shown to very closely mimic each other [16]. Furthermore, android area in DXA includes liver, pancreas and lower part of the heart, and fat accumulation in these areas is associated with metabolic abnormalities, even more closely than the accumulation of visceral fat [30].

## Conclusions

This first long-term study supports the acute protein balance studies showing that adding carbohydrates to postexercise protein ingestion may not have large effect on the RT adaptations. Whey proteins, however, increased abdominal fat loss and relative fat-free mass adaptations in response to resistance training when compared to fast-acting carbohydrates. Therefore, if the main goal is to maximize fat loss responses to RT especially from abdominal area without compromising increases in muscle hypertrophy, whey protein instead of carbohydrates can be recommended for the postexercise nutrition.

## Abbreviations

REE: Resting energy expenditure
1 RM: One repetition maximum
ANOVA: Analysis of variance
CHO: Carbohydrates
CSA: Cross-sectional area
DXA: Dual-energy X-ray absorptiometry
FFM: Fat-free mass
FM: Fat mass
HDL: High-density lipoprotein
HS: Hypertrophy and strength
ICC: Intraclass correlation coefficient
LDL: Low-density lipoprotein
MS: Maximal-strength
MVC: Maximal voluntary contraction
PS: Power-strength
QF: Quadriceps femoris
RT: Resistance training
SHP: Strength, power and hypertrophy
